# Supplementary material for: Scalable method for micro-CT analysis enables large scale quantitative characterization of brain lesions and implants
Source: Sci Rep. 2020 Nov 30;10:20851. doi: 10.1038/s41598-020-77796-3 (PMC7705725; doi:10.1038/s41598-020-77796-3)
Supplement: Supplementary file 2 — Supplementary Information 2. [file 41598_2020_77796_MOESM2_ESM.pdf]

Supplementary Information

Scalable method for micro-CT analysis enables large scale quantitative characterization of brain lesions and implants

David B. Kastner[1,2‡*], Viktor Kharazia[2‡], Rhino Nevers[2], Clay Smyth[2], Daniela A. Astudillo-Maya[2], Greer M. Williams[1], Zhounan Yang[1], Cristofer M. Holobetz[1], Luca Della Santina[3,4], Dilworth Y. Parkinson[5], Loren M. Frank[1,2,6]

[1]*Department of Psychiatry and Behavioral Sciences, University of California, San Francisco, CA 94143, USA*

[2]*Kavli Institute for Fundamental Neuroscience and Department of Physiology, University of California, San Francisco, CA 94158, USA*

[3]*Deparment of Ophthalmology, University of California, San Francisco, CA 94143, USA*

[4]*Bakar Computational Health Science Unit, University of California, San Francisco, CA 94158, USA*

[5]*Advanced Light Sources Division, Lawrence Berkeley National Labs, Berkeley, CA 94720, USA*

[6]*Howard Hughes Medical Institute*

[‡]*These authors contributed equally to this work*

[*]*Corresponding author:* David B. Kastner, david.kastner2@ucsf.edu

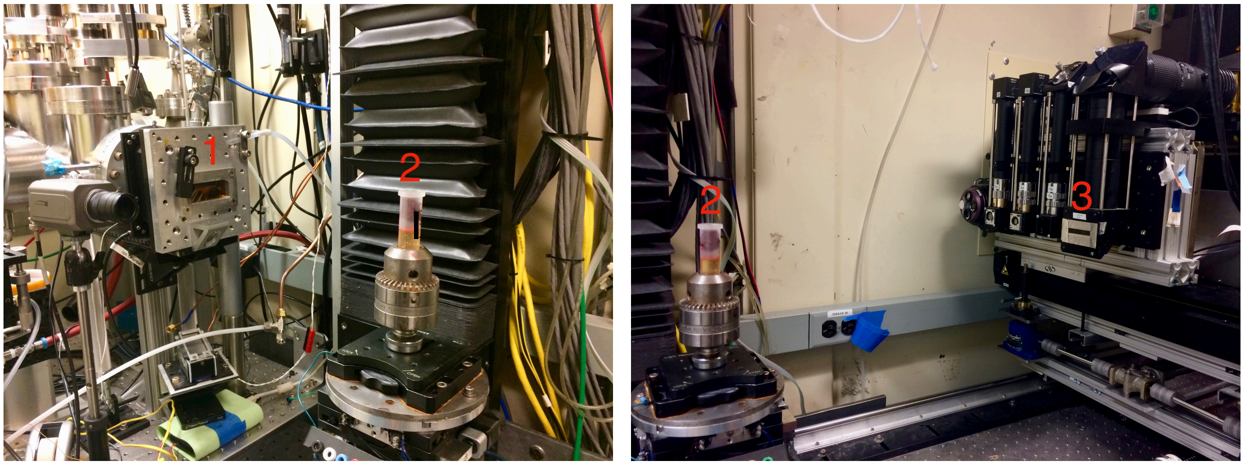

Figure S1: Layout of micro-CT at the ALS. 1 indicated the location of the x-ray source. 2 indicated the location of the sample, which rotates. 3 indication the location of the collector optics.

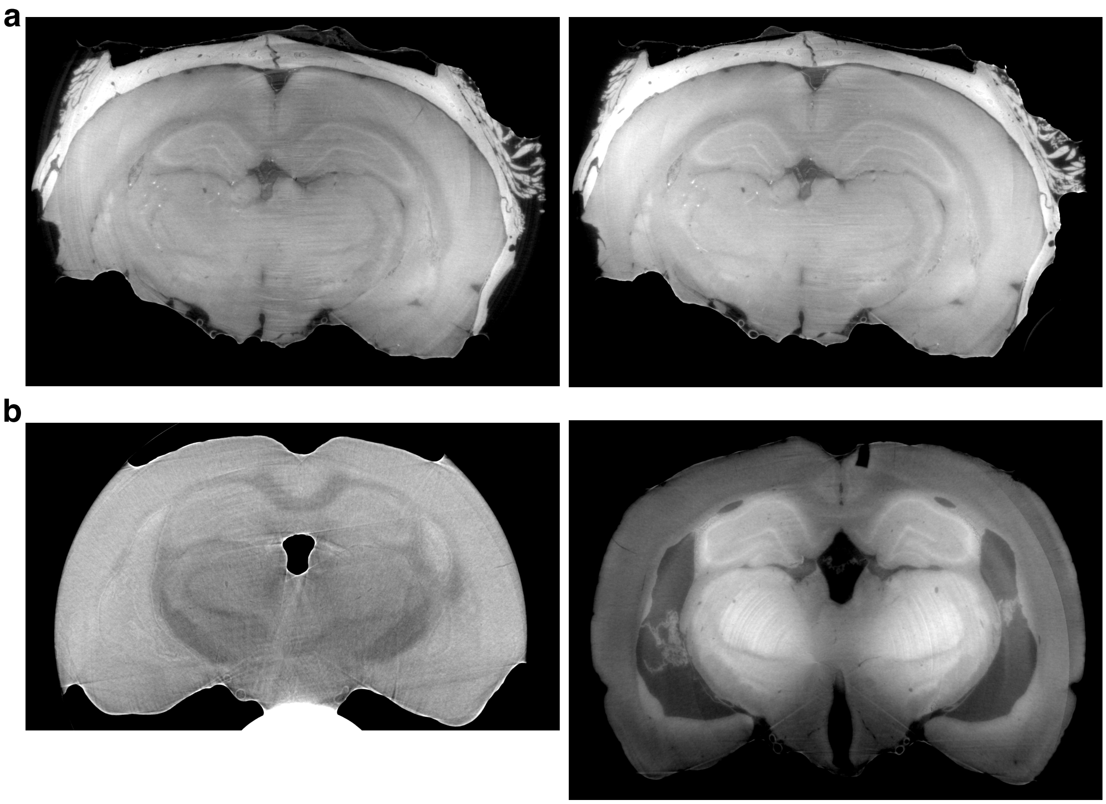

Figure S2: a) The same brain was scanned twice, ~2 hours apart. First (left) and second (right) scans show comparable image quality, indicating no detectable evidence for degradation of the sample due to the radiation dose used. **b**) A brain process without eosin staining (left) and a different brain for comparison with eosin staining. These brains were processed without the skull to maximize the potential for contrast without eosin staining. Anatomical structure can be visualized without eosin, but the eosin provides higher contrast. For scanning each brain, energy levels were independently set to optimize transmitted x-rays. For the brain without eosin an energy level of 20 keV was used. For the brain with eosin an energy level of 22 keV was used.

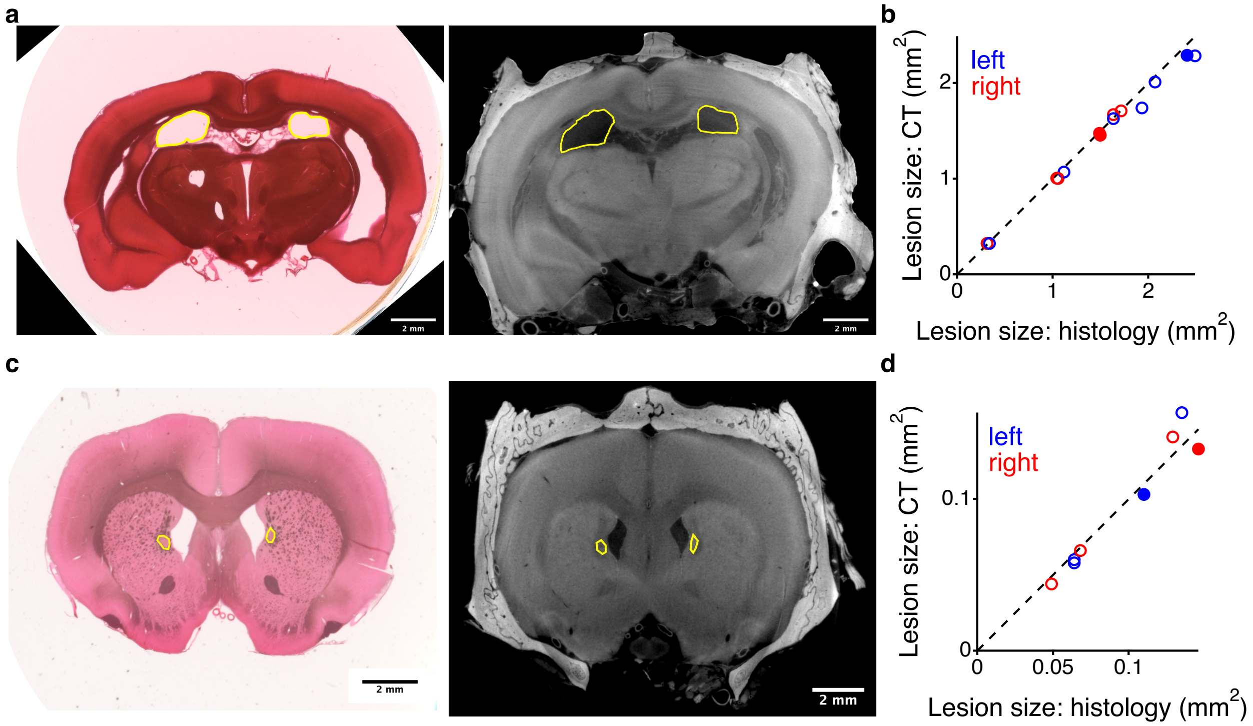

Figure S3: Lesion size validation. **a**) Example histological slice (right) and corresponding virtual CT slice (left) from a brain that underwent hippocampal lesioning. For the histology, holes were created in the middle of the brain to enable identification of the left side of the brain. **b**) Comparison of hippocampus lesion areas for all slices within the brain. Red indicates hippocampal lesions from the right side of the brain. Blue indicates hippocampal lesions from the left side of the brain. Filled in circles indicate the lesion areas from the slices shown in a panel **a**. **c**) Example histological slice (right) and corresponding virtual CT slice (left) from a brain that underwent DMS lesioning. **d**) Comparison of DMS lesion areas for all slices within the brain. Red indicates lesions from the right side of the brain. Blue indicates lesions from the left side of the brain. Filled in circles indicate the lesion areas from the slices shown in a panel **c**. For the histology, no additional staining was added to the brains, and the imaging was done with the slices free floating in PBS to minimize distortions. For **a** & **c** CT slice was chosen to best match the histological slice. Lesion boundaries (yellow borders) were traced in Fiji and the area within the boundary was calculated. For **b** & **d** dotted line plots the identity line for comparison.

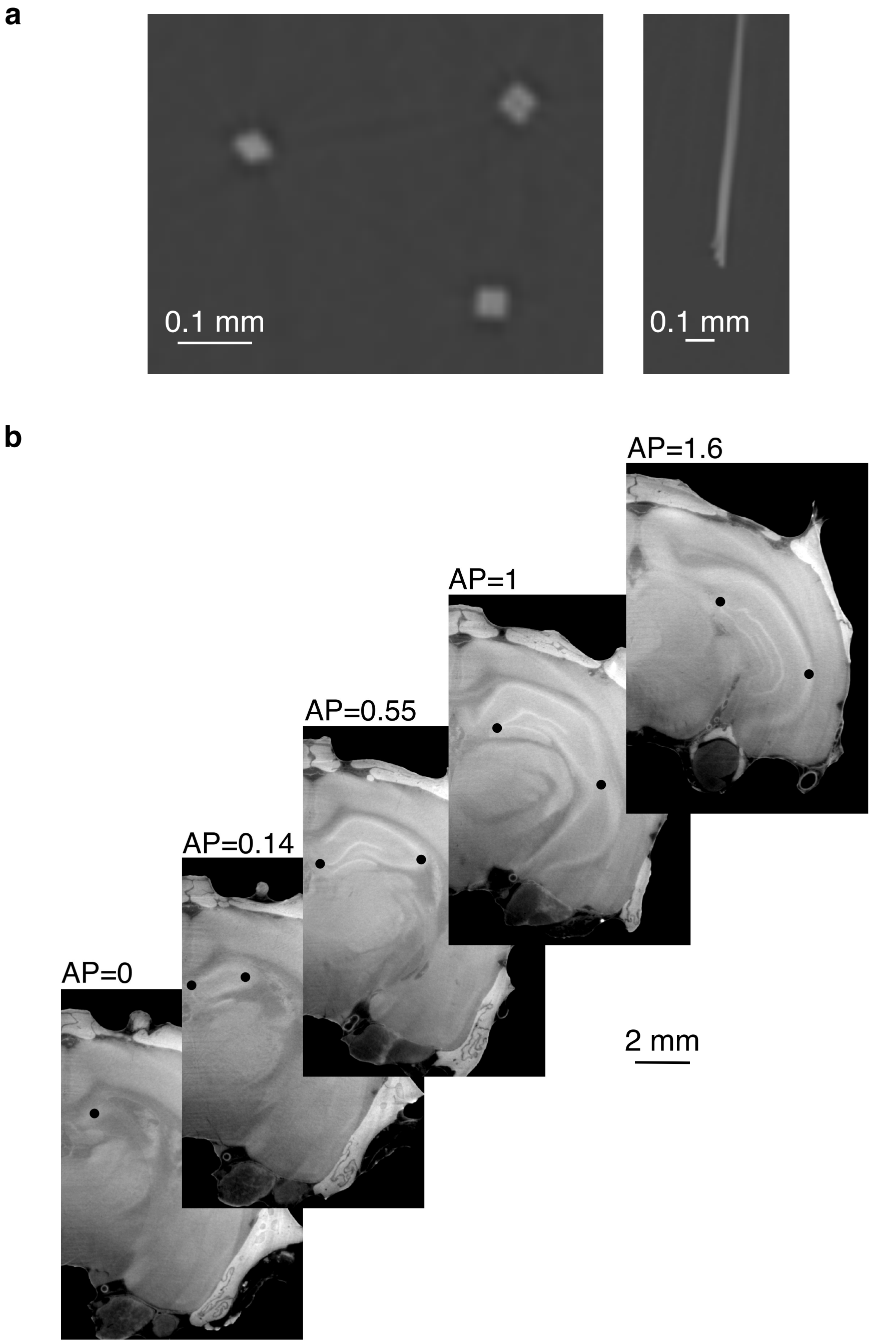

Figure S4: a) Fine scale tetrode structure can be visualized with the micro-CT. Left shows a virtual horizontal slice, where the four individual electrodes that make up a single tetrode can be seen. Right show a virtual coronal slice, where the individual electrode and the braiding of the tetrode can be seen. **b**) Example brain showing the location of the coordinates the make up the two-dimension space from Fig 4C. Virtual coronal slices of an example brain, with sample points of the two-dimensional space demarcated as black dots. At AP=0 the dot shows the origin point. At AP=1 the rightmost dot shows the point (1,1). For the rest of the slices the rightmost dot shows the medial part of the dentate cell layer and the leftmost dot shows the lateral aspect of the hippocampal cell layer. Scale bar applies to all images within the panel.

Video 1: Video showing the entire three-dimensional rendering of the same brain from Fig 1 and virtually slicing the brain along the sagittal, coronal and horizontal axes.
